# Comment on the role of interferons in the pathology of beta cell destruction in type 1 diabetes

**DOI:** 10.1007/s00125-024-06264-8

**Published:** 2024-09-07

**Authors:** Sigurd Lenzen

**Affiliations:** Institute of Experimental Diabetes Research, Hannover Medical School, Hannover, Germany

**Keywords:** Aetiopathology, Pancreatic beta cell destruction, Proinflammatory cytokines, Therapeutic perspectives, Type 1 diabetes mellitus

*To the Editor:* I comment on the original publication ‘Interferons are key cytokines acting on pancreatic islets in type 1 diabetes’ by Coomans de Brachène et al [1].

Based upon in vitro studies using the RNA-seq methodology, the authors conclude in this publication that IFN-α and IFN-γ had a greater impact on the beta cell transcriptome when compared with IL-1β and TNF-α [1]. Thereby, they imply that interferons, in particular IFN-γ, are of a greater importance in the pathology of pancreatic beta cell dysfunction and death in type 1 diabetes mellitus than TNF-α and IL-1β. This is an inadequate conclusion, and in particular is misleading, since it might indicate that IFN-γ is a primary target for therapies with curative potential aiming to reverse autoimmunity in the treatment of type 1 diabetes. It is agreeable that IFN-γ is a proinflammatory cytokine that is very toxic for beta cells [2]. In human type 1 diabetes, however, the proinflammatory cytokines that are crucially responsible for beta cell dysfunction and death are IL-1β and, in particular, TNF-α, rather than IFN-γ, which plays only a subordinate role. This is because IFN-γ is only weakly expressed at both the gene and protein levels in the immune cell infiltrate of pancreatic islets in humans with type 1 diabetes [3, 4]. This proinflammatory cytokine pattern (high levels of TNF-α and IL-1β and a low level of IFN-γ) is also found in the immune cell infiltrate of the islets of different animal models of type 1 diabetes, not only in the NOD mouse but also in the biobreeding (BB) rat and the LEW.1AR1-iddm (IDDM) rat models [3]. It is also the case in the pancreas of humans with late autoimmune diabetes in adults (LADA), a milder form of autoimmune diabetes, as well as in the IDDM rat model of LADA [4]. In LADA, the expression of TNF-α is less prominent relative to that of IL-1β, explaining the less aggressive beta cell toxicity [4]. This conclusion is supported by a wealth of experimental evidence collected during recent years, detailed below.

Crucial support comes from the anti-CD3 antibody ‘disease modifying’ therapy, approved by the U.S. Food and Drug Administration in November 2022, which can delay the manifestation of type 1 diabetes by 2 to 3 years [5]. When administered in combination with an anti-TNF-α antibody, such a therapy even opens up the prospect of a long-term therapeutic effect with curative potential. The successful implementation of such a therapy is based on numerous studies over the last years, which have shown (not cited in the article [1]) that the proinflammatory cytokine TNF-α plays a central role in the destruction of pancreatic beta cells in the pathogenesis of type 1 diabetes [3, 6].

Combination therapy with anti-CD3 and anti-TNF-α eliminates the infiltration of immune cells expressing proinflammatory cytokines [6, 7], which cannot be achieved in a combination therapy with anti-IFN-γ [6]. An exclusive reference to an antidiabetic effect of anti-IFN-γ in the NOD mouse model of type 1 diabetes, as the authors do in their publication [1], is insufficient. A large number of studies in the NOD mouse have shown therapeutic success in this type 1 diabetes mouse model using a wide variety of preventive therapies, but none of these therapies could be successfully transferred to the situation in patients with type 1 diabetes [8]. A successful transfer of such a therapeutic concept into an effective translational therapy for patients with type 1 diabetes, which enables a return to a normal metabolic state, is therefore not recognisable based on the facts presented in Coomans de Brachène et al’s publication [1], at least not in the foreseeable future. Therefore, the conclusion is that IFN-γ does not play a central role in the pathogenesis of type 1 diabetes; such a concept lacks the necessary experimental basis. The concept by Coomans de Brachène et al [1] cannot provide a viable approach for the establishment of a successful therapeutic approach to tackle type 1 diabetes.

In the pathogenesis of type 1 diabetes in humans and in the IDDM rat model, a high level of IL-1β in the immune cell infiltrate is a prominent feature of the disease process [6, 7]. Crucial, however, is the generation of a second proinflammatory cytokine, namely TNF-α [6, 7], which is generated in a high amount in the immune cell infiltrate and which, in conjunction with IL-1β, is responsible for the process of beta cell destruction [6, 7]. Expression of IL-1β alone in the infiltrating immune cells in the pancreatic islets primarily inhibits beta cell function, as documented by the pathology in the Cohen rat model of autoimmune diabetes [9], so that anti-IL-1β therapy alone is successful there [10].

An important weakness of this in vitro study is the fact that only single proinflammatory cytokines were compared, as the authors admit in the discussion section [1], while no combination of TNF-α and IL-1β, which optimally mirrors the situation in the human and IDDM rat model type 1 diabetes pancreas [6, 7], was tested, which the senior author also used in early in vitro studies [11].

Therefore, in conclusion, a proinflammatory cytokine that is weakly expressed in the immune cell infiltrate in the pancreas of humans with type 1 diabetes, such as IFN-γ, cannot be the proinflammatory cytokine primarily responsible for beta cell destruction in type 1 diabetes, and can therefore also not be the target for a therapeutic strategy, as documented by the ineffectiveness of an anti-IFN-γ antibody therapy [6].

## Abbreviation

IDDM: LEW.1AR1-iddm
